# The Prognostic Value of Inflammatory Indices in Acute Pulmonary Embolism

**DOI:** 10.3390/diagnostics15030312

**Published:** 2025-01-29

**Authors:** Mihai Ștefan Cristian Haba, Oana Mădălina Manole, Ana Maria Buburuz, Ionuț Tudorancea, Irina-Iuliana Costache-Enache, Viviana Onofrei

**Affiliations:** 1 Department of Internal Medicine, Faculty of Medicine, University of Medicine and Pharmacy ”Grigore T. Popa”, 700115 Iasi, Romania; 2 Department of Cardiology, “St. Spiridon” County Clinical Emergency Hospital, 700111 Iasi, Romania; 3 Department of Physiology, Faculty of Medicine, University of Medicine and Pharmacy ”Grigore T. Popa”, 700115 Iasi, Romania

**Keywords:** pulmonary embolism, PESI, NLR, NLPR, SII, SIRI

## Abstract

**Background:** Acute pulmonary embolism (PE) is a condition with increased morbidity and mortality. It is important to identify patients with high mortality risk. Inflammation and thrombosis are interconnected in the pathophysiology of PE. The aim of the study was to investigate the prognostic value of multiple blood cellular indices such as neutrophil-to-lymphocyte ratio (NLR), lymphocyte-to-monocyte ratio (LMR), platelet-to-lymphocyte ratio (PLR), neutrophil-to-lymphocyte platelet ratio (NLPR), systemic immune–inflammation index (SII), systemic inflammation response index (SIRI) and aggregate index of systemic inflammation (AISI) in acute PE. **Methods**: A total of 157 patients with acute PE confirmed by chest computed tomographic angiography (CTPA) were enrolled. These patients were divided into two categories according to the simplified pulmonary embolism severity index (sPESI): high risk and low risk. **Results**: Univariate logistic regression analysis showed that right ventricle dysfunction, NLR, SII and SIRI were significantly associated with high risk of acute PE. NLR of 4.32 was associated with high-risk PE with a sensitivity of 57.4% and specificity of 65.7% (AUC = 0.635). SII of 1086.55 was associated with high-risk PE with a sensitivity of 55.7% and specificity of 71.4% (AUC = 0.614). SIRI of 2.87 was associated with high-risk PE with a sensitivity of 59% and specificity of 62.9% (AUC = 0.624). Multivariate logistic regression analysis demonstrated that right ventricle dysfunction, NLR, PLR and NLPR are independent predictors of high-risk acute PE. Secondly, NLR, NLPR, SII and SIRI were significantly correlated with in-hospital mortality of acute PE. Based on receiver-operating characteristic (ROC) curve values of 7.66 for NLR (AUC 0.911, sensitivity of 85.7% and sensibility of 83%), 0.02 for NLPR (AUC 0.871, sensitivity of 85.7% and sensibility of 70%), 1542.71 for SII (AUC 0.782, sensitivity of 71.4% and sensibility of 72%) and 5.72 for SIRI (AUC 0.788, sensitivity of 71.4% and sensibility of 73%) could predict in-hospital mortality. **Conclusions**: The blood cellular indices (NLR, NLPR, SII and SIRI) are associated with high-risk acute PE and in-hospital mortality. Right ventricular dysfunction, NLR and NLPR are independent predictors for high-risk acute PE.

## 1. Introduction

Venous thromboembolism (VTE) is the third leading cause of death worldwide after acute myocardial infarction and stroke [1]. The incidence is about 1–2 per 1000 people but varies with age, increasing to 11.2 per 1000 people for people over 80 years [2], and region, with a higher incidence in Africa, between 0.7 and 61%, and a lower incidence, 0.2 per 1000 person-years, in Asia [3]. The pathophysiology of VTE is complex, based on Virchow’s triad described in 1856—hypercoagulability, venous stasis and endothelial dysfunction [1]. Pulmonary embolism (PE) is a life-threatening manifestation of VTE and a medical emergency, with significant mortality and morbidity greater than deep venous thrombosis [4]. Mortality from acute PE was 5–9% at 30 days and nearly 20% at 1 year [5,6]. About 8% of people over 45 develop PE during their lifetime [7].

Because of the low specificity and sensitivity of the clinical picture in PE, the development of clinical probability scores (Wells and Geneva scores) [8] in conjunction with the value of D-dimer and age-adjusted D-dimer [8], respectively, points to the usefulness and interpretation of imaging techniques [9]. The diagnosis of PE is based on imaging methods, such as computed tomographic angiography (CTPA) and planar ventilation–perfusion lung scintigraphy. The increased availability of these techniques leads to overdiagnosis of segmental and subsegmental PE [5,10]. The clinical and prognostic impact of these phenotypes is not well known, but current practice might lead to unnecessary anticoagulant treatment or even fibrinolytic treatment with a higher risk of bleeding [9,11,12].

PE is unpredictable; hence, risk scores, such as the Pulmonary Embolism Severity Index (PESI) and later sPESI, have been developed to evaluate the severity [13,14]. The sPESI score—based on six of the eleven variables of the original score—showed similar prognostic accuracy and clinical value to the original one [13,14]. The prognostic value has also been shown for the severity of PE of cardiac troponin [15], brain natriuretic peptide (BNP), N-terminal pro-brain natriuretic peptide (NT-proBNP) [8,16], heart-type fatty acid binding protein (H-FABP), right ventricle (RV) dysfunction [9], inflammatory cytokines (interleukine-4, IL-6, IL-8, IL-10, IL-1α, IL-1β) [17], oxygen reactive species and myeloperoxidase [18]. Other research has continued to evaluate more accessible and cost-effective predictors of prognosis of acute PE.

Thrombosis and inflammation are interconnected and play a central role in the development of PE [19,20]. Under inflammatory conditions, the vascular endothelium changes its conformation from antithrombotic to prothrombotic. Leucocytes, especially neutrophils and monocytes, facilitate the initiation of the coagulation cascade [20]. Neutrophils release cytokines, histones and neutrophil extracellular traps (NETs) that contribute to thrombosis. Il-6 and tumor necrosis factor-alpha (TNF-α) activate tissue factors and inhibit plasminogen activators [21]. Histones bind the von Willebrand factor and inhibit thrombomodulin, activating platelets [19]. NETs facilitate endothelial cell degradation and apoptosis, activating platelets and the coagulation cascade [22]. Platelets secrete immunomodulatory molecules and modulate the immune response. They display receptors with an immunizing role, such as toll-like receptors (TLRs), complement receptors, antibody receptors (FcRs) and intracellular NOD-like receptors (NLRs) [23]. Neutrophils, monocytes and platelets interact with each other and with the vascular endothelium and play a critical role in the development of VTE [20].

**Figure 1:**
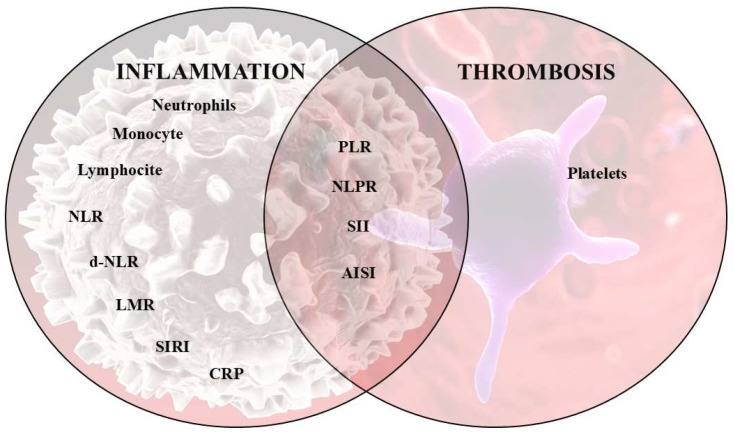
The interaction between inflammation and thrombosis.

Inspired by the interplay between thrombosis and inflammation, recent studies have explored the usefulness of blood cellular indices in various conditions. These indicators are readily available in the blood panel and are accessible and cost-effective. The neutrophil-to-lymphocyte ratio (NLR) and platelet-to-lymphocyte ratio (PLR) are better markers of systemic inflammation than white blood cell (WBC) count in clinical practice [24]. Previous studies have shown the ratios have great predictive potential in cancers [25] and cardiovascular diseases [26]. PLR has been correlated with the severity of atherosclerosis [27], being an indicator of coronary disease [28] and critical limb ischemia in peripheral arterial disease [29]. A meta-analysis demonstrated that increased values of NLR could be used as a marker for patient mortality/MACEs in acute coronary syndrome patients [30]. Also, high NLR levels were associated with an increased incidence of in-hospital death in patients with atrial fibrillation [31]. In a cohort study, SIRI and SII were associated with both in-hospital mortality and long-term mortality in patients with chronic heart failure. SIRI had a similar prognostic value in predicting in-hospital mortality compared with NT-proBNP and a higher prognostic value in predicting both in-hospital and 3-year mortality compared with C-reactive protein (CRP) [32]. In a 20-year follow-up cohort study of 42,875 adults, it was demonstrated that higher values of SII and SIRI were associated with cardiovascular mortality and all-cause mortality and are both independent risk factors for them [33]. SIRI and SII were also evaluated in coronary artery disease [34] (Figure 1).

Studies have demonstrated the predictive potential of blood cellular indices in PE [14,35,36,37]. Previous research has evaluated one or two indicators, with the varying nature of markers used or study objectives [38,39,40]. NLR has been the most intensively studied blood cellular indicator in PE—approximately thirty studies have focused on it, while SII and SIRI have been examined in a few studies.

The complex pathophysiology of PE requires the evaluation of markers from multiple cell lines to obtain a comprehensive view of the inflammatory and coagulation status. In our study, we aimed to evaluate the association of multiple blood cellular indices (NLR, d-NLR, LMR, PLR, NLPR, SII, SIRI, AISI) for both inflammation and thrombosis with severity, prognosis, and in-hospital mortality assessed in acute PE.

## 2. Materials and Methods

### 2.1. Study Design

**Figure 2:**
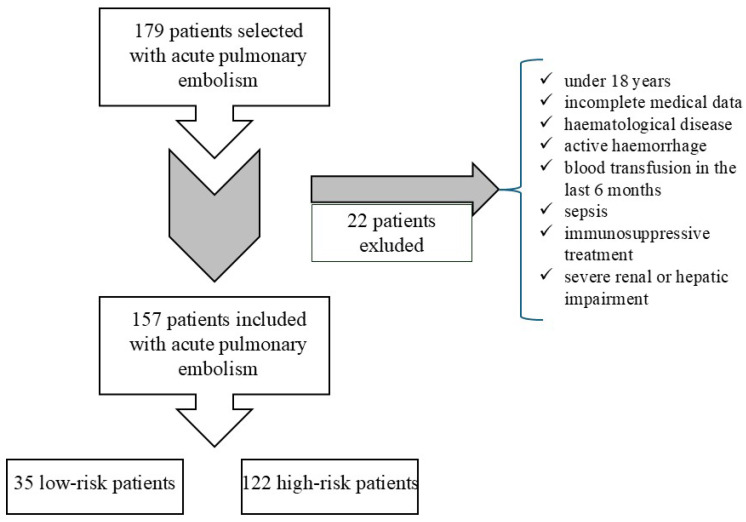
The study design flowchart.

We included 179 patients hospitalized for acute PE between January 2020 and August 2023 in the Cardiology Department of “Sf. Spiridon” Hospital, Iasi. The inclusion criteria were age ≥ 18 years old, confirmed diagnosis of acute PE, complete medical data and patient consent. Twenty-two patients were excluded from the study according to the exclusion criteria, as shown in Figure 2. A total of 157 patients were finally included in the study (Figure 2). The clinical manifestations of patients varied; most of them had dyspnea. Unfortunately, the exact time between the onset of symptoms and the presentation time cannot be quantified because most patients presented when their symptoms worsened and not when they started.

### 2.2. Paraclinical Measurements

Blood samples were taken within the first hour of presentation and were analyzed immediately using the same apparatus in the Hematology and Biochemistry Departments. At the time the blood samples were collected, no patient had fever, symptoms of cold/infection or Severe acute respiratory syndrome Coronavirus 2 (SARS-CoV2) infection. The estimated glomerular filtration rate (eGFR) was calculated according to the 2021 CKD-EPI formula. The inflammatory markers used are illustrated in Table 1.

Electrocardiogram was performed within the first 10 min of the patient’s presentation in the Emergency Department using a BTL-08 LC PLUS electrocardiograph device (BTL Industries Ltd., Cleveland Way, Stevenage, Hertfordshire, UK). Echocardiography was performed at admission using a General Electric Vivid ^TM^ V7 ultrasound device (General Electric, Boston, CA, USA) to evaluate PE parameters. Right ventricle (RV) dysfunction was defined as: T-wave inversion in V1–V4 leads, QR pattern in V1, S1Q3T3 pattern or right bundle branch block on electrocardiogram and/or right ventricle (RV) dilatation with basal RV/left ventricle proportion more than 1, impaired free wall contractility in comparison to its apex (McConnel sign), reduced tricuspid annular plane systolic excursion (TAPSE) measured with M-mode (<16 mm) and reduced peak systolic (S′) velocity of tricuspid annulus (<9.5 cm/s).

The diagnosis of acute PE was established according to the European Society of Cardiology (ESC) Guidelines (ESC, 2019) [8] by CTPA within 3–6 h of presentation, which identified partial or complete filling defects in the pulmonary arteries in at least two consecutive sections.

The sPESI score (age > 80 years, history of cancer, history of chronic cardiopulmonary disease, pulse rate ≥ 110/min, systolic blood pressure < 100 mmHg, arterial blood oxygen saturation < 90%) was calculated based on the patient’s clinical data at presentation. An sPESI score of 0 was considered a low sPESI score (low-risk acute PE), while an sPESI score ≥1 was considered a high sPESI score (high-risk acute PE).

### 2.3. Ethics

Ethical approval was obtained from the Ethics Committee of the “Grigore T. Popa” University of Medicine and Pharmacy Iasi and the Ethics Committee of “St. Spiridon” Clinical Emergency Hospital and was conducted according to the Helsinki Declaration. All patients signed an informed consent statement, which mentioned that the results would be used for research purposes.

### 2.4. Statistical Analysis

Descriptive statistics were reported as frequencies and percentages for categorical variables and median and lower–upper quartile for continuous variables. The statistical analysis of data was performed using IBM SPSS Statistics version 26.0 (IBM, Armonk, NY, USA). The normality of the distribution for continuous data was evaluated using the Kolmogorov–Smirnov test. The Mann–Whitney U test was performed to compare continuous variables without a normal distribution, Student’s *t*-test for continuous variables with a normal distribution and Chi-square was used to compare categorical variables. For correlation, we conducted a Spearman correlation. Furthermore, a receiver–operating characteristic (ROC) curve analysis was performed to identify the sensibility and specificity of blood cellular indices. A univariate and multivariate logistic regression was conducted. *p* < 0.05 was considered statically significant.

## 3. Results

A total of 157 acute PE patients, from 179 patients enrolled, were included in the study after applying the exclusion criteria. We identified 122 patients (77.7%) with high-risk PE and 35 patients (22.3%) with low-risk PE. Eighty-five patients were male (53.1%), and seventy-two were female (45%), with no significant difference between the two groups. The median age of the patients from the high-risk group was 68 years, which was significantly higher than the low-risk group (59 years, *p* = 0.001). There was no difference in the hospitalization period between high-risk and low-risk group(eight days). Although there were more high-risk patients presenting with central or trunk involvement pulmonary embolism (93 high-risk patients vs. 22 low-risk patients, respectively, 12 high-risk patients vs. 2 low-risk patients), it was not statistically significant (*p* = 0.11, *p* = 0.45, respectively). One hundred and four patients with high-risk acute PE overall (124 patients) were significantly associated with RV dysfunction (*p* = 0.0001). The thrombolytic treatment was applied in 23 patients, 21 with high-risk acute PE and only two with low-risk acute PE, without statistical significance (*p* = 0.09). There was no bleeding in the group of patients who received thrombolytic treatment. There were seven non-survivor patients (five of them received thrombolytic treatment), all presented with high-risk acute PE (*p* = 0.05). The results are presented in Table 2.

The etiology of PE has been varied (Table 3). The association of deep vein thrombosis was significantly higher in the high-risk group (42 high-risk patients vs. 20 low-risk patients, *p* = 0.01). Paraneoplastic PE was only in patients belonging to the high-risk group (*p* = 0.008). Thrombophilia and oral contraceptive use were only found in low-risk PE patients (*p* = 0.01). There were no statistically significant differences between the comorbidities of patients in the two groups (Table 4).

CRP, neutrophil count, NLR, dNLR, NLPR, SII and SIRI were all significantly elevated in high-risk acute PE patients (all *p* < 0.05). In the high-risk group, WBC count and AISI were higher, while lymphocyte count, platelet count and LMR were lower, but neither was significantly different from the low-risk group. The patients in the high-risk PE group showed a degree of kidney disease (eGFR of 73.5 mL/min/1.73 m^2^ vs. 91 mL/min/1.73 m^2^, *p* = 0.002) (Table 5).

As seen in Table 6, all studied blood cellular indices were correlated with CRP. NLR, dNLR, NLPR, SII and SIRI were significantly associated with both original and simplified PESI scores and in-hospital mortality. LMR correlated only with PESI and sPESI scores. All blood cellular indices, except for NLR and NLPR, significantly correlated with pulmonary infarction. 

**Figure 3:**
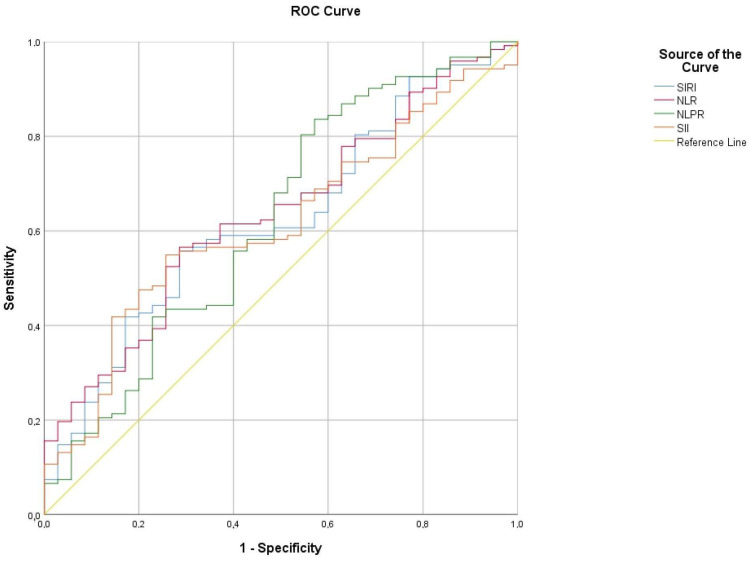
ROC curve indicating sensitivity and specificity of NLR, NLPR, SII and SIRI to predict high-risk acute PE.

Based on receiver–operating characteristic curve (ROC) analysis, the NLR, NLPR, SII and SIRI cut-off value of admission for prediction of a high sPESI score was determined as 4.32, 0.011, 1086.55 and 2.87, respectively, with a sensitivity of 57.4%, 81.1%, 55.7% and 59%, respectively, and specificity of 65.7%, 42.9%, 71.4% and 62.9%, respectively (Table 7 and Figure 3).

**Figure 4:**
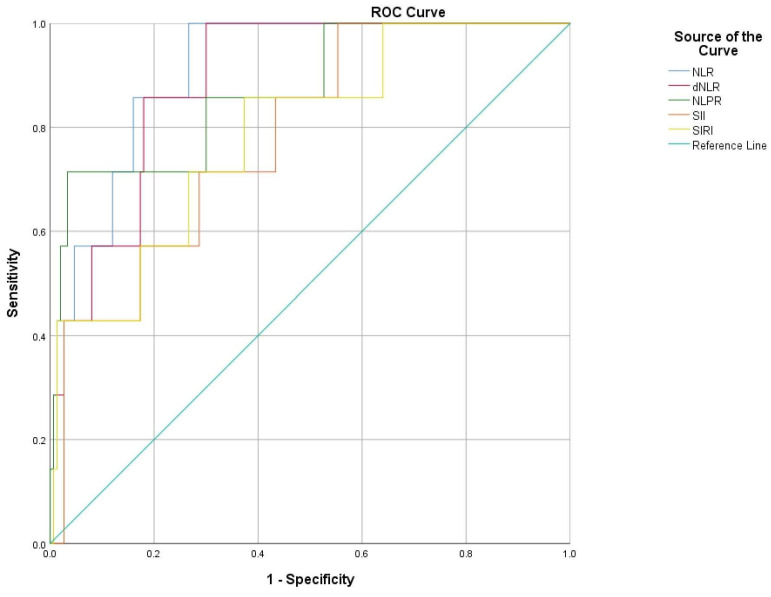
ROC curve indicating sensitivity and specificity of NLR, d-NLR, NLPR, SII and SIRI to predict in-hospital mortality in acute PE.

In-hospital mortality based on ROC curve analysis can be predicted with a cut-off value of 7.66 for NLR (AUC 0.911, sensitivity of 85.7% and sensibility of 83%), 6.99 for dNLR (AUC 0.890, sensitivity of 85.7% and sensibility of 82%), 0.02 for NLRP (AUC 0.871, sensitivity of 85.7% and sensibility of 70%), 1542.71 for SII (AUC 0.782, sensitivity of 71.4% and sensibility of 72%) and 5.72 for SIRI (AUC 0.788, sensitivity of 71.4% and sensibility of 73%) (Table 8 and Figure 4).

Univariate analysis showed that neutrophil count, RV dysfunction, NLR, dNLR, SII and SIRI were all correlated with a high sPESI score (Table 9). The multivariate logistic regression model showed that NLR, NLPR, PLR and RV dysfunction were independent predictors of high-risk acute PE (Table 10).

## 4. Discussion

In this study, we aimed to investigate the predictive value of blood cellular indices in high-risk PE patients and in-hospital mortality. We demonstrated that NLR, dNLR, NLPR, SII and SIRI are significantly correlated with high-risk acute PE. NLR, NLPR and PLR are independent predictors. NLR, NLPR, SII and SIRI correlated with in-hospital mortality in acute PE.

We found that age was significantly increased in high-risk patients, probably due to the associated comorbidities that influence the prognosis of acute PE. Deep vein thrombosis, the main cause of acute PE, was more prevalent in the high-risk group, suggesting the role of thrombotic burden. Even if in-hospital mortality did not demonstrate statistical significance, the non-survivors were all from the high-risk group (*p* = 0.05). High-risk PE significantly presented with RV dysfunction, in concordance with the literature [8].

In our study, several basic hematological parameters, such as platelet count and WBC count, did not show any statistical difference between low-risk and high-risk PE groups, similar to previous studies [41]. However, the patients in the sPESI ≥ 1 group (high-risk group) presented elevated neutrophil count. These results mirrored the results of Karakurt and collaborators [42], who found an increased level of neutrophils in patients diagnosed with PE. Furthermore, increased levels of neutrophils were correlated with high-risk PE, similar to several previous studies [43,44].

NLR has been the most intensively studied blood cellular indicator in PE [36]. In our research, NLR was correlated with both original and simplified PESI scores and was an independent predictor for high-risk acute PE and in-hospital mortality. Previous studies demonstrated the diagnostic [45,46] and prognostic value of NLR in acute PE [35,47]. Telo and collaborators [44] found that NLR was associated with a high sPESI score in 82 patients with acute PE, while Kose and collaborators [46] reported the association between NLR and low- and moderate–low-risk PE but not with moderate–high- and high-risk PE. In our study group, we identified a cut-off value NLR of 4.32 to predict high-risk acute PE with a sensibility of 57.4% and a specificity of 65.7%. Our results are similar to other researchers [44], who found a lower cut-off value of NLR 3.56 correlated with sPESI ≥ 1 score with a comparable AUC of 0.675, a sensibility of 66% and a specificity of 53% [44]. If we refer to the previous cut-off value of NLR (3.5), the sensitivity would increase slightly (65.6%), but specificity would decrease to 45.7 in our case. NLR has also been reported to be an independent predictor of massive PE [38].

Phan and collaborators [47] demonstrated the positive correlation between NLR and original and simplified PESI scores and found higher values of NLR in non-survivor PE patients. In a meta-analysis that included seven retrospective studies, it was found that an elevated NLR is associated with an 8.43% higher risk of overall mortality and a 10.13% higher risk of short-term mortality [35]. Ma and collaborators [39] have shown that NLR is an independent predictor of 30-day mortality, and the risk increased by 13% with the increase of one unit for NLR. Our study was able to illustrate the positive correlation between NLR and in-hospital mortality, with a cut-off value of 7.66 to predict it with a sensibility of 85.7% and a specificity of 83%. Other studies reported lower cut-off NLR values associated with all cause-mortality in acute PE (5.46), with a lower sensibility of 75% and a specificity of 66.9% in 191 patients with PE [47] and 5.5 in 228 patients with acute PE [48]. If we refer to the previous cut-off value of NLR (5.5) to predict mortality in acute PE, the sensitivity would increase to 100%, while the specificity would decrease to 65.3% in our case. The association between NLR and long-term mortality in PE has been illustrated in several other studies [49,50,51,52]. However, a few studies have reported that there is no significant association between NLR and non-survivor patients with PE [53,54]. Ertem and collaborators [53] did not find a significant difference in NLR between survivors and non-survivors in 294 patients with PE. Also, Ghaffari and collaborators [54] showed no correlations between NLR and non-survivors in 492 patients with PE.

Another blood cellular indicator is PLR, which includes the coagulation pathway and provides additional information on the interaction between inflammation and thrombosis in PE. A large number of studies have included PLR in the assessment of diagnosis, severity and short- and long-term mortality in patients with PE [36]. Our study results were consistent with other research [38,44,45]. Other studies have demonstrated the diagnostic [38,45] and prognostic value of PLR in PE [35,41,43,44,46,48], with different cut-off values associated with mortality [43,44,47]. Contrarily, there is research that could not obtain a significant association between PLR and the occurrence of VTE [55,56,57,58,59] or could not predict mortality in PE [39,60,61].

By adding platelets to the NLR, another marker, NLPR, is obtained that evaluates both inflammatory and coagulation pathways. In our study, we found that NLPR correlated with both original PESI and s-PESI in patients with PE. Multivariate logistic regression analysis demonstrated that NLPR could be interpreted as an independent prognostic marker for high-risk acute PE. A cut-off optimal value of 0.011 could predict high-risk acute PE with a sensibility of 81.1% and a specificity of 42.9%. More than that, NLPR was associated with in-hospital mortality and could be predicted with a sensibility of 85.7% and a specificity of 70% for a cut-off value of 0.02. To our knowledge, NLPR has not been studied in PE before.

Monocyte-related indicators were also evaluated in past studies, such as simply monocyte count (LMR) or monocyte–lymphocyte ratio (MLR) [36], with contradictory results on mortality in PE [46,53,59]. In our study, the value of LMR was decreased in high-risk PE patients but without statistical significance.

SII and SIRI are two novel accessible markers that simultaneously assess various parameters, such as platelet count, neutrophil count, lymphocyte count and monocyte count. Their utility in predicting adverse events in inflammatory diseases [62,63], cancers [64,65] and cardiovascular diseases [33,66,67] has been proven. We demonstrated that SII and SIRI were correlated with high-risk acute PE and in-hospital mortality and can predict both. Few studies evaluated SII and SIRI in diagnosis and severity in PE. Gok and collaborators [68] have found increased SII levels in patients with PE, proportional to thrombotic burden. They also found higher SII levels in in-hospital non-survivor patients. Another study demonstrated increased values of SII in patients with VTE [59]. A recent study also demonstrated the predictive value for intermediate-risk PE of both SII (cut-off 705.6) and SIRI (cut-off 1.59) with a sensibility of 75.31% and 82.72%, respectively, and specificity of 71.26% and 68.97%, respectively [40]. In our study, we found that a cut-off value of 1542.71 for SII and 5.72 for SIRI could predict in-hospital mortality of patients with acute PE with a sensibility of 71.4% for both and a specificity of 72% and 73%, respectively.

AISI is another marker used to assess inflammation in COVID-19. One study evaluating the development of venous thromboembolism in patients with COVID-19 demonstrated that elevated AISI values were associated with the development of thromboembolic complications [69]. In our study, we did not obtain any significant correlations.

CRP is a well-known protein associated with acute inflammation. Our results showed its increased values in patients with elevated PESI and sPESI scores and its correlation with blood cellular indices, which is in agreement with other studies [41,70].

We only found a correlation between PLR and patients who received thrombolytic treatment; because many hemodynamically unstable patients who received fibrinolytic treatment in the emergency department did not survive, they were not included in this research.

In some cases, PE is accompanied by pulmonary infarction and may be clinically expressed. Pulmonary infarction is ischemia followed by necrosis of the lung parenchyma secondary to pulmonary artery occlusion [71]. To define pulmonary infarction, there are three perspectives: clinical (association between pleuritic chest pain and hemoptysis), imaging (pleural-based consolidations, atelectasis, pleural effusion) and histologic (area of “coagulation necrosis”) [71,72]. The lung is an organ with dual circulation, bronchial and pulmonary, with anastomoses present between them, but they are not functional under normal healthy conditions. In the case of distal pulmonary artery obstruction (frequently a segmental or subsegmental ram, less than 3 mm), the increased pressure of blood flow from the collaterals from the bronchial circulation exceeds the capacity of the obstructed pulmonary circulation to accommodate. This, together with increased endothelial permeability (secondary to capillary ischemia and inflammation), leads to extravasation of erythrocytes in the alveola. The inability to reabsorb (inadequate blood flow) hemoglobin within 1–2 days causes necrosis of the lung parenchyma and, thus, pulmonary infarction with the development of fibrotic scarring at that level [71,72]. Pulmonary infarction has been associated with young age (absence of collaterals), smoking and heart disease, but studies on associated biomarkers are limited [73]. The study area of pulmonary infarction is ideal for the development of an important inflammation process [72]. In our study, we obtained significantly higher values of PLR, SII, SIRI, AISI, dNLR and LMR in patients with pulmonary infarction.

Our study evaluated the prognostic value of blood panel-derived cellular indices. They are inexpensive, affordable and accessible to any laboratory, with immediately available results. They can provide information about severity from the time of PE diagnosis and can help stratify patients according to their evolution.

There are some limitations in our study. It was a retrospective study in a single center with a small number of patients and did not evaluate laboratory results at different times (before diagnosis of PE and follow-up). We were not able to compare our results with other predictors, such as troponin, NT-pro-BNP or H-FABP, because they were processed on different devices. Blood sample analysis was performed in the hematology and biochemistry laboratory and not with point-of-care tests.

Further research is necessary to evaluate the association between these inflammatory indices and pulmonary artery pressure or blood gas values. It would also be interesting to validate point-of-care devices that could provide these cellular indices derived from the blood count already calculated.

## 5. Conclusions

In conclusion, this study demonstrated that NLR, NLPR, SII and SIRI are associated with high-risk PE patients. NLR, NLPR and PLR are independent predictors for high-risk PE. NLR, NLPR, SII and SIRI are also associated with in-hospital mortality in PE. The integration of blood panel-derived indices with already known parameters (hemodynamic variables, ultrasound parameters) can help to assess the prognosis of acute PE.

## Figures and Tables

**Table 1:** Blood cellular indices and their formulae used in the study.

Blood Cellular Indices	Formula
Neutrophil-to-lymphocyte ratio	NLR = $\frac{neutrophil\;count}{lymphocyte\;count}$
Derived-NLR	d-NLR = $\frac{neutrophil\;count}{white\;blood\;cells\;total\;count-neutrophil\;count}$
Lymphocyte-to-monocyte ratio	LMR = $\frac{lymphocyte\;count}{monocyte\;count}$
Platelet-to-lymphocyte ratio	PLR = $\frac{platelet\;count}{lymphocyte\;count}$
Neutrophil-to-lymphocyte, platelet ratio	NLPR = $\frac{neutrophil\;count}{lymphocyte\;count\times platelet\;count}$
Systemic immune—inflammation index	SII = $\frac{platelet\;count\;x\;neutrophil\;count}{lymphocyte\;count}$
Systemic inflammation response index	SIRI = $\frac{neutrophil\;count\times monocyte\;count}{\;lymphocyte\;count}$
Aggregate index of systemic inflammation	AISI = $\frac{neutrophil\;count\;x\;monocyte\;count\times platelet\;count}{lymphocyte\;count}$

**Table 2:** Baseline characteristics of the studied patients.

	Overall (*n* = 157) (100%)	Low-Risk sPESI = 0 (*n* = 35) (22.3%)	High-Risk sPESI ≥ 1 (*n* = 122) (77.7%)	*p*
Baseline characteristics				
Age ^a^ (years)	68 (53–75.5)	59 (46–69)	68 (57.00–76.25)	**0.001**
Gender ^b^ Male, *n* (%)	85 (53.1%)	17 (48.6%)	68 (55.7%)	0.45
Hospitalization ^a^ (days)	8 (7–10)	8 (6–9)	8 (7–11)	0.07
RV dysfunction ^b^, *n* (%)	124 (79.0%)	20 (57.1%)	104 (85.2%)	**0.0001**
Central pulmonary embolism ^b^, *n* (%)	115 (73.2%)	22 (62.9%)	93 (76.2%)	0.11
Pulmonary trunk involvement ^b^, *n*(%)	14 (8.9%)	2 (5.7%)	12 (9.8%)	0.45
Pulmonary infarction ^b^, *n* (%)	19 (12.1%)	4 (11.4%)	15 (12.3%)	0.89
Thrombolytic treatment ^b^, *n* (%)	23 (14.6%)	2 (5.7%)	21 (17.2%)	0.09
In-hospital mortality ^b^, *n* (%)	7 (4.5%)	0 (0.0%)	7 (5.7%)	0.05
History of COVID-19 infection ^b^, *n* (%)	26 (16.6%)	5 (14.3%)	21 (17.2%)	0.68

COVID-19 = Coronavirus Disease 2019. Mann–Whitney U test for continuous variables without normal distribution and Chi-square test for categorical variables. Statistically significant values were indicated in bold. ^a^ Median (lower quartile–upper quartile). ^b^ Number (frequency).

**Table 3:** Etiology of the acute PE of the studied patients.

	Overall (*n* = 157) (100%)	Low-Risk sPESI = 0 (*n* = 35) (22.3%)	High-Risk sPESI ≥ 1 (*n* = 122) (77.7%)	*p*
Etiology				
Deep vein thrombosis ^b^, *n* (%)	62 (39.5%)	20 (57.1%)	42 (34.4%)	**0.01**
Neoplasia ^b^, *n* (%)	21 (13.4%)	0 (0.0%)	21 (17.2%)	**0.008**
Bone fracture ^b^, *n* (%)	5 (3.2%)	2 (5.7%)	3 (2.5%)	0.33
Immobilization ^b^, *n* (%)	33 (21.0%)	9 (25.7%)	24 (19.7%)	0.43
Thrombophilia ^b^, *n* (%)	3 (1.9%)	3 (8.6%)	0 (0%)	**0.001**
Oral contraceptives ^b^, *n* (%)	3 (1.9%)	3 (8.6%)	0 (0%)	**0.001**
Chronic venous insufficiency ^b^, *n* (%)	44 (28.0%)	13 (37.1%)	31 (25.4%)	0.17
Antiphospholipid syndrome ^b^, *n* (%)	1 (0.6%)	1 (2.9%)	0 (0.0%)	0.06

Chi-square test for categorical variables. Statistically significant values indicated in bold. ^b^ Number (frequency).

**Table 4:** Comorbidities of the studied patients.

	Overall (*n* = 157) (100%)	Low Risk sPESI = 0 (*n* = 35) (22.3%)	High Risk sPESI ≥ 1 (*n* = 122) (77.7%)	*p*
Comorbidities				
Smoking ^b^, *n* (%)	39 (24.8%)	10 (28.6%)	29 (23.8%)	0.56
BMI ≥ 30 kg/m^2 b^, *n* (%)	18 (11.5%)	6 (17.1%)	12 (9.8%)	0.23
High blood pressure ^b^, *n* (%)	86 (54.8%)	21 (60.0%	65 (53.3%)	0.48
Dyslipidemia ^b^, *n* (%)	46 (29.3%)	14 (40.0%)	32 (26.2%)	0.11
Diabetes ^b^, *n* (%)	26 (16.6%)	3 (8.6%)	23 (18,9%)	0.14
Atrial fibrillation ^b^, *n* (%)	15 (9.6%)	4 (11.4%)	11 (9.0%)	0.66
Ischemic heart disease ^b^, *n* (%)	12 (7.6%)	3 (8.6%)	9 (7.4%)	0.81
Chronic heart failure ^b^, *n* (%)	34 (21.7%)	4 (11.4%)	30 (24.6%)	0.09
Chronic kidney disease ^b^, *n* (%)	31 (19.7%)	3 (8.6%)	28 (23.0%)	0.06
Silicosis ^b^, *n* (%)	1 (0.6%)	0 (0.0%)	1 (0.8%)	0.59

BMI = body mass index. Chi-square test for categorical variables. ^b^ Number (frequency).

**Table 5:** Laboratory parameters of the studied patients.

	Overall (*n* = 157) (100%)	Low-Risk sPESI = 0 (*n* = 35) (22.3%)	High-Risk sPESI ≥ 1 (*n* = 122) (77.7%)	*p*
Laboratory parameters				
CRP ^a^ (mg/dL)	3.91 (1.48–9.94)	3.09 (0.79–5.78)	4.39 (1.73–12.03)	**0.03**
WBC count ^a^ (×10^3^/µL)	9.93 (6.84–11.87)	8.27 (6.08–11.63)	10.18 (7.23–10.22)	0.10
Neutrophil count ^a^ (×10^3^/µL)	7.12 (4.51–9.29)	5.99 (3.77–8.38)	7.33 (5.00–9.42)	**0.04**
Eosinophil count ^a^ (×10^3^/µL)	0.08 (0.02–0.15)	0.10 (0.06–0.16)	0.07 (0.02–0.15)	0.06
Basophil count ^a^ (×10^3^/µL)	0.03 (0.02–0.05)	0.02 (0.02–0.04)	0.03 (0.02–0.05)	0.38
Lymphocyte count ^a^ (×10^3^/µL)	1.48 (1.12–2.21)	1.72 (1.18–2.40)	1.44 (1.10–2.04)	0.08
Monocyte count ^a^ (×10^3^/µL)	0.77 (0.58–1.01)	0.65 (0.57–0.92)	0.81 (0.59–1.05)	0.15
NLR ^a^	4.56 (2.83–7.04)	3.64 (2.57–5.63)	5.01 (2.91–7.60)	**0.01**
dNLR ^a^	4.39 (2.05–6.63)	3.57 (1.45–5.25)	4.63 (2.15–7.07)	**0.02**
LMR ^a^	2.13 (1.37–3.14)	2.80 (1.55–3.69)	2.01 (1.36–2.94)	0.05
Platelet count ^a^ (×10^3^/µL)	231 (175.50–290.50)	234 (171–322)	230 (179.75–286.75)	0.66
PDW ^a^	12.10 (11.15–14.05)	12 (10.30–14.70)	12.15 (11.20–13.92)	0.62
MPV ^a^ (fL)	10.40 (9.85–11.10)	10.60 (9.80–11.30)	10.40 (9.90–11.10)	0.99
PCT ^a^ (%)	0.24 (0.19–0.29)	0.24 (0.18–0.33)	0.23 (0.19–0.29)	0.79
PLR ^a^	152.03 (102.60–235.19)	144.64 (101.81–206.10)	155.53 (102.85–236.67)	0.40
NLPR ^a^	0.01 (0.01–0.03)	0.01 (0.007–0.02)	0.02 (0.01–0.03)	**0.02**
AISI ^a^	768.26 (317.81–1570.07)	562.45 (308.74–997.34)	840.20 (317.82–1627.84)	0.08
SII ^a^	1086.15 (562.54–1763.16)	954.22 (455.49–1174.82)	1149.77 (581.97–1876.21)	**0.04**
SIRI ^a^	3.16 (1.59–6.38)	2.56 (1.22–4.13)	3.55 (1.78–6.84)	**0.02**
Creatinine ^a^ (mg/dL)	0.87 (0.72–1.1)	0.82 (0.69–0.96)	0.89 (0.73–1.17)	**0.03**
eGFR ^a^ (mL/min/1.73 m^2^)	79.00 (63.00–96.00)	91 (78.00–103.00)	73.50 (61.00–92.00)	**0.002**
Cholesterol ^a^ (mg/dL)	175.00 (144.00–198.00)	171.50 (142.75–195.50)	174.00 (144.50–200.00)	0.97
LDL cholesterol ^a^ (mg/dL)	121.00 (97.75–138.75)	121.00 (95.5–138.75)	121.00 (98.50–138.50)	0.91
Triglycerides ^a^ (mg/dL)	119.50 (90.00–141.25)	110.00 (83.25–138.50)	122.50 (94.25–142.75)	0.25
Uric acid ^a^ (mg/dL)	4.80 (3.60–7.00)	5.06 (3.60–7.15)	4.70 (3.55–7.00)	0.68

AISI = aggregate index of systemic inflammation, CRP = C-reactive protein, LMR = lymphocyte-to-monocyte ratio, MCV = mean cell volume, MPV = mean platelet volume, NLPR = neutrophil-to-lymphocyte-platelet ratio, NLR = neutrophil-to-lymphocyte ratio, PCT = plateletcrit, PDW = platelet distribution width, PLR = platelet-to-lymphocyte ratio, SII = systemic immune–inflammation index, SIRI = systemic inflammation response index, WBC = white blood cells. Mann–Whitney U test for continuous variables without normal distribution and Student’s t-test for continuous variables with normal distribution (eGFR, Cholesterol). Statistically significant values were indicated in bold. ^a^ Median (lower quartile–upper quartile).

**Table 6:** Correlations between blood cellular indices and severity scores; demographic and paraclinical parameters.

	NLR		dNLR		LMR		PLR		NLPR		AISI		SII		SIRI	
	*p*	coeff	*p*	coeff	*p*	Coeff	*p*	coeff	*p*	coeff	*p*	coeff	*p*	coeff	*p*	coeff
sPESI	0.001	0.26	0.003	0.23	0.03	−0.16	0.33	0.07	0.001	0.25	0.06	0.14	0.02	0.18	0.01	0.20
PESI	0.0001	0.27	0.004	0.22	0.01	−0.19	0.30	0.08	0.0001	0.29	0.07	0.14	0.03	0.17	0.007	0.21
Age	0.08	0.14	0.48	0.05	0.82	−0.01	0.01	0.20	0.25	0.09	0.83	0.01	0.05	0.15	0.97	0.003
Hospitalization	0.39	0.06	0.18	0.10	0.54	−0.04	0.52	−0.05	0.34	0.07	0.44	0.06	0.72	0.02	0.32	0.07
CRP	0.0001	0.37	0.0001	0.42	0.0001	−0.33	0.04	0.16	0.003	0.24	0.0001	0.44	0.0001	0.37	0.0001	0.47
Central pulmonary embolism	0.79	0.02	0.64	0.03	0.70	0.03	0.03	−0.17	0.10	0.12	0.16	−0.11	0.19	−0.10	0.98	−0.001
Pulmonary infarction	0.07	0.14	0.005	0.22	0.01	−0.20	0.01	0.19	0.58	−0.04	0.0001	0.32	0.0001	0.28	0.002	0.24
Thrombolytic treatment	0.26	0.08	0.07	0.14	0.35	−0.07	0.04	−0.15	0.06	0.14	0.67	0.03	0.69	−0.03	0.11	0.12
In-hospital mortality	0.0001	0.29	0.0001	0.27	0.77	−0.02	0.32	0.07	0.001	0.26	0.08	0.13	0.01	0.20	0.01	0.20

**Table 7:** Optimal cut-off levels of blood cellular indices to predict high-risk acute PE.

	*p*-Value	AUC	Sensitivity	Specificity
NLR ≥ 4.32	0.01	0.635	57.4%	65.7%
NLPR ≥ 0.011	0.02	0.624	81.1%	42.9%
SII ≥ 1086.55	0.04	0.614	55.7%	71.4%
SIRI ≥ 2.87	0.02	0.624	59%	62.9%

**Table 8:** Cut-off optimal levels of blood cellular indices for in-hospital mortality in acute PE.

	*p*-Value	AUC	Sensitivity	Specificity
NLR ≥ 7.66	0.0001	0.911	85.7%	83%
dNLR ≥ 6.99	0.001	0.890	85.7%	82%
NLPR ≥ 0.02	0.001	0.871	85.7%	70%
SII ≥ 1542.71	0.01	0.782	71.4%	72%
SIRI ≥ 5.72	0.01	0.788	71.4%	73%

**Table 9:** Univariate logistic analysis of paraclinical parameter predictors of high-risk acute PE.

	*p*-Value	Odds Ratio	95% CI
Neutrophil count	0.03	1.15	1.01–1.31
Platelet count	0.42	0.99	0.99–1.00
RV dysfunction	0.001	0.23	0.10–0.53
NLR	0.01	1.19	1.03–1.38
dNLR	0.01	1.19	1.03–1.36
LMR	0.15	0.85	0.69–1.05
PLR	0.60	1.00	0.99–1.00
SII	0.03	1.00	1.00–1.00
SIRI	0.03	1.16	1.01–1.32
AISI	0.07	1.00	1.00–1.00

**Table 10:** Multivariate logistic regression analysis of independent predictors of high-risk acute PE.

	*p*-Value	Odds Ratio	95% CI
RV dysfunction	0.0001	0.17	0.06–0.46
NLR	0.01	2.98	1.24–7.20
NLPR	0.01	0.00	0.00–0.00
PLR	0.03	0.98	0.97–0.99
LMR	0.17	0.76	0.51–1.13
SIRI	0.44	0.88	0.65–1.20
WBC	0.85	1.09	0.41–2.86
Neutrophil count	0.57	0.69	0.20–2.42
Platelet count	0.67	1.00	0.99–1.01
CRP	0.42	1.02	0.96–1.10

Model verification: ANOVA (*p* = 0.001).

## Data Availability

The data presented in this study are available from the corresponding author upon request. The data are not publicly available due to the confidentiality of personal data.
